# The effects of Reiki practice on breast cancer patients: a systematic review

**DOI:** 10.1007/s00520-026-10889-3

**Published:** 2026-06-18

**Authors:** Zeliha Turan, Derya Tülüce

**Affiliations:** 1 Department of Obstetrics and Gynecology Nursing, Faculty of Health Sciences, Osmanbey Campus, 63300 Haliliye-Şanliurfa, Turkey; 2 Department of Nursing, Faculty of Health Sciences, Osmaniye Korkut Ata University, Osmaniye, Turkey

**Keywords:** Breast cancer, Symptom management, Reiki, Systematic review, Complementary therapies, Supportive care

## Abstract

**Purpose:**

Breast cancer and its treatments cause multifaceted symptoms in women, affecting their physical, psychological, and emotional well-being. Complementary practices such as Reiki are increasingly used to support symptom management, particularly during chemotherapy. This systematic review aims to evaluate the effects of Reiki practice on improving symptoms in breast cancer patients within the framework of Martha E. Rogers’ “Science of Unitary Human Beings.”

**Method:**

This study was conducted as a systematic review in accordance with the PRISMA 2020 guidelines. A comprehensive literature search was performed in the PubMed, Web of Science, CINAHL (EBSCOhost), Google Scholar, and DergiPark databases, with the search updated through May 2026 prior to the final analysis. Randomized controlled trials, experimental studies, and quasi-experimental studies investigating Reiki interventions in breast cancer patients were included. Methodological quality and risk of bias were independently assessed by two researchers using Joanna Briggs Institute (JBI) critical appraisal tools and the Cochrane Risk of Bias Tool.

**Results:**

Four studies meeting the inclusion criteria included a total of 339 participants. The included studies suggested that Reiki practice may reduce fatigue and improve overall comfort and well-being. Some studies also reported improvements in quality of life, comfort, mental well-being, and mood.

**Conclusion:**

Reiki appeared to be a safe and well-tolerated complementary intervention in the included studies. Reiki practice may contribute to symptom management, improve quality of life, and support psychological well-being in breast cancer patients. However, due to the limited number of studies, small sample sizes, and methodological differences, larger, well-designed randomized controlled trials are needed.

## Introduction

Breast cancer is one of the most common types of cancer among women worldwide, causing significant challenges for patients not only physically but also psychologically and emotionally [1–3]. Both the disease itself and standard treatment methods used in cancer therapy, such as surgery, chemotherapy, radiotherapy, and hormone therapy, lead to multifaceted side effects including pain, fatigue, sleep disturbances, anxiety, stress, and a decrease in quality of life [2, 4, 5]. These effects mean that patients need not only medical but also holistic care approaches [2, 5, 6].

Complementary and integrative medicine methods are increasingly being preferred by individuals with cancer in response to this need [7–10]. Among these methods, Reiki is a non-invasive energy therapy practice based on the principle of transferring universal life energy to the body through the hands. Various studies have shown that Reiki has effects such as reducing stress, promoting relaxation, alleviating pain and anxiety, and improving sleep quality [6, 11–16]. Reiki treatments are reported to be particularly effective in breast cancer patients as a tool for symptom management and improving quality of life [6, 17–20].

In this context, Reiki stands out as a method that addresses both physical and psychological symptoms with a holistic approach. Indeed, Martha E. Rogers’ Science of Unitary Human Beings, a nursing theory, defines the human being as a whole in constant energy exchange with its environment and relates health to the balance of the individual’s energy field. According to Rogers, the aim of nursing is to strengthen the interaction between the individual and their environment to enhance human unity and harmony, and to broaden communication patterns between energy sources to realize the maximum potential of health [21–23]. Since Reiki is conceptually described as aiming to balance an individual’s energy field, it can be considered a complementary nursing intervention that is consistent with Rogers’ theoretical framework. The fundamental concepts in Rogers’ theory, including energy field, pattern, resonance, integrality, and balance, may conceptually align with the principles underlying Reiki practices [22, 24]. In this context, Reiki aims not only to alleviate symptoms but also to support the holistic health of the individual [24–27].

This systematic review aims to evaluate the effects of Reiki practices in individuals with breast cancer and to interpret these effects by integrating them with the perspective of holistic nursing theory, known as Martha E. Rogers’ Science of Unitary Human Beings, often referred to in the literature as the “Energy Field Theory.”

## Methods

### Research question

The research question of this systematic review study is structured using the PICO approach:

- Population (P): Patients diagnosed with breast cancer
- Intervention (I): Breast cancer patients who received Reiki practice
- Comparisons (C): Individuals not receiving Reiki/individuals receiving sham Reiki/those receiving standard care/those receiving other complementary care
- Outcomes (O): Physical symptoms, psychological symptoms, quality of life

*Accordingly, the research question is:* Does Reiki practice have an effect on improving the symptoms of breast cancer patients?

- Is Reiki practice effective on physical symptoms in patients diagnosed with breast cancer?
- Is Reiki practice effective in reducing psychological symptoms in breast cancer patients?
- Is Reiki practice effective in improving the quality of life of breast cancer patients?

### Research design

This study was conducted as a systematic review to evaluate the effects of Reiki practice on symptom management in breast cancer patients. The review was conducted and reported in accordance with the Preferred Reporting Items for Systematic Reviews and Meta-Analyses (PRISMA) 2020 guidelines [28]. The literature search was updated in May 2026 prior to the final analysis and reporting of the review. Randomized controlled trials, experimental studies, and quasi-experimental studies investigating Reiki interventions in breast cancer patients were considered eligible for inclusion. Methodological quality assessment and study selection were conducted independently by the researchers.

### Protocol

In the study, the recommendations in Cochrane Manual version 6.2 were followed [29], and the Preferred Reporting Items for Systematic Reviews (PRISMA) 2020 checklist was used [28]. The protocol for this systematic review was registered in the International Prospective Register of Systematic Reviews (PROSPERO) (Registration No: CRD420261391817).

### Literature review

The articles included in this study were searched in the PubMed, Web of Science, CINAHL (EBSCOhost), Google Scholar, and DergiPark databases. In addition to database searches, gray literature sources including OpenGrey, ProQuest Dissertations & Theses, conference proceedings, ClinicalTrials.gov, and the Turkish National Thesis Center were also searched. However, no additional eligible studies meeting the inclusion criteria were identified.

The search strategy was developed using Medical Subject Headings (MeSH) terms and free-text keywords related to Reiki and breast cancer. The main search terms included: [Reiki] OR [Reiki therapy] OR [energy healing] OR [biofield therapy] OR [complementary therapy] AND [breast cancer] OR [breast neoplasms] OR [breast carcinoma] AND [quality of life] OR [fatigue] OR [anxiety] OR [pain] OR [supportive care]. Studies published from database inception through May 2026 were considered, without publication year restrictions. Language restrictions were applied to include studies published in English or Turkish only. Randomized controlled trials and experimental and quasi-experimental studies were eligible for inclusion.

### Inclusion criteria

In this study, inclusion criteria were defined according to PICOS [29]:

- Population (P): Patients diagnosed with breast cancer
- Intervention (I): Reiki practice
- Comparisons (C): Individuals not receiving Reiki/individuals receiving sham Reiki/those receiving standard care/those receiving other complementary care
- Outcomes (O): To evaluate the effectiveness of Reiki practice in breast cancer patients, physical, psychological, or quality-of-life outcomes should be assessed using a valid measurement tool, clinical evaluation method, or biophysiological measurement device
- Study designs (S): Randomized controlled trials (RCTs), experimental and quasi-experimental studies

### Exclusion criteria

- Descriptive research
- Research where full text is unavailable
- Research conducted in languages other than English or Turkish
- Research that received a “poor” score on the quality assessment tool
- Research involving energy therapies other than Reiki (e.g., healing touch, therapeutic touch, acupuncture)
- Research examining the practice of Reiki in individuals with cancer types other than breast cancer
- Qualitative research that evaluates the practice of Reiki focusing solely on the experiences of healthcare professionals

**Fig. 1:**
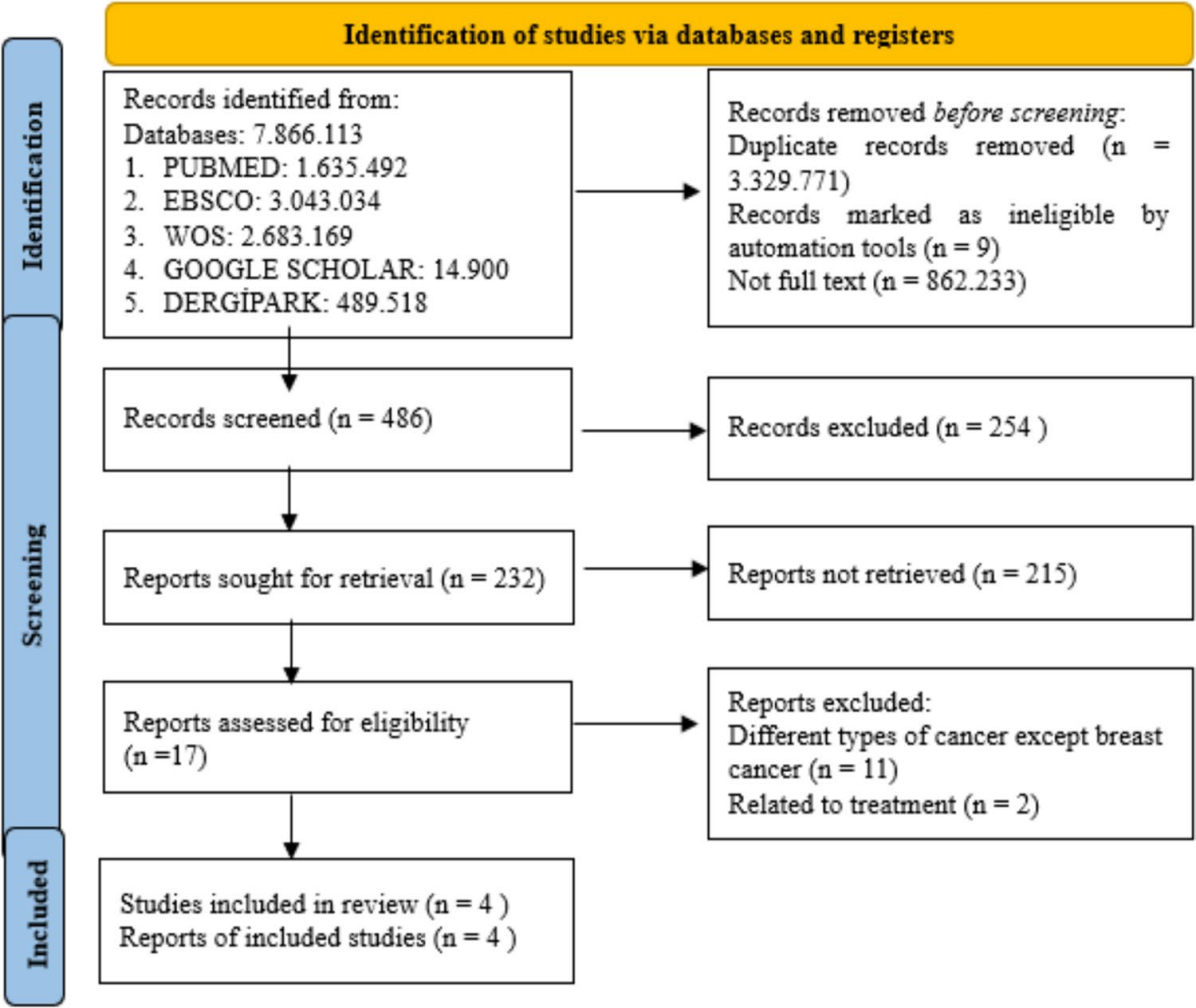
PRISMA 2020 flow diagram

To reduce the risk of possible bias in the study, the literature search, article selection, data extraction, and full texts of the studies were carried out independently by the researchers in detail. The studies that were found as a result of the search and met the inclusion criteria are shown in the PRISMA 2020 Flow Diagram [28] (Fig. 1).

### Bias risk assessment

Risk of bias of the included studies was independently assessed by two authors (author 1, author 2) using Cochrane Risk of Bias assessment approaches appropriate to the study designs [30]. The risk of bias was divided into three categories: (1) low risk, (2) uncertain, and (3) high risk. The authors then met to check their bias assessments in accordance with the guidelines, and no disagreements or inconsistencies were found between the authors.

### Cochrane bias risk criteria [30]

- Bias arising from the randomization process (selection bias)
- Bias due to deviations from the intended interventions (performance bias)
- Bias in measuring the outcome (determination bias)
- Bias arising from incomplete outcome data (missing bias)
- Bias in selecting the reported outcome (reporting bias)

### Quality assessment

Methodological quality assessment of the studies included in the research was carried out by two independent researchers using Joanna Briggs Institute (JBI) critical assessment checklists according to the selected research types. In this study, JBI quality assessment tools and checklists, whose Turkish validity and reliability were established by Nahcivan and Seçginli (2017), were used [31].

In the JBI-MASTARI checklists, each item is evaluated with 1 point for “Yes,” and 0 points for “No,” “Not specified,” and “Not applicable.” The MASTARI Critical Assessment score for the “Experimental and Quasi-Experimental Research Checklist” ranges from 0 to 10 points. A higher total score indicates higher methodological quality. The lowest methodological quality assessment score was 5, and the highest was 9.

## Results

### Study selection and characteristics

The number of participants in the studies included in this systematic review [6, 17, 19, 32] ranged from 36 to 189. The total number of participants was 339. Two of the studies were randomized controlled trials: one was a quasi-experimental study, and the other was an experimental study with unspecified randomization. The countries where the studies were conducted were the USA (2), Turkey (1), and India (1).

The Reiki interventions varied across studies in terms of session duration, frequency, practitioner experience, and comparison groups. Reiki sessions ranged from 20 to 42 min and were generally administered during chemotherapy sessions by trained Reiki practitioners. Comparison groups included standard care (control), sham Reiki, companion support, or no intervention. Outcome measures mainly focused on fatigue, symptom distress, mood, comfort, well-being, and quality of life.

### Effects of Reiki on physical symptoms

Two studies reported improvements in physical symptoms following Reiki interventions [6, 19]. Karaman and Tan (2021) found significant reductions in fatigue and general symptom burden among participants receiving Reiki during chemotherapy compared with the control group [6]. Improvements were observed in symptoms including fatigue, pain, insomnia, nausea-vomiting, dyspnea, appetite loss, and overall functioning.

Similarly, Kuril (2020) reported reductions in chemotherapy-related discomfort and perceived improvements in physical, emotional, and mental well-being after Reiki sessions [19]. Participants described relief from treatment-related side effects and improved overall comfort following Reiki interventions.

However, findings were not entirely consistent across studies. Orsak et al. (2015) did not report statistically significant improvements in symptom distress among patients receiving Reiki compared with standard care or companion support [17].

### Effects of Reiki on psychological outcomes

Several studies suggested that Reiki interventions may positively influence psychological well-being and emotional comfort [6, 17, 19, 32]. Improvements were reported in mental comfort, relaxation, mood, vitality, and general emotional well-being following Reiki interventions.

Orsak et al. (2015) reported improvements in overall mood and vitality among patients receiving Reiki compared with standard care, although companion support demonstrated stronger effects in several psychological outcomes [17]. Similarly, Catlin et al. (2011) found improvements in mental comfort and mental well-being following both real Reiki and sham Reiki interventions [32]. The findings from sham-controlled studies suggest that therapeutic attention, supportive interaction, relaxation, or placebo-related mechanisms may contribute to the perceived benefits associated with Reiki interventions.

### Effects of Reiki on quality of life

Three studies reported improvements in quality of life or general well-being following Reiki interventions [6, 17, 32]. Karaman and Tan (2021) observed significant improvements in general well-being and functional status among participants receiving Reiki during chemotherapy [6]. Likewise, Orsak et al. (2015) found that quality-of-life scores were higher in Reiki and companion support groups compared with standard care [17].

Catlin et al. (2011) also demonstrated improvements in comfort and well-being among participants receiving Reiki-related interventions compared with standard care [32]. Nevertheless, no significant differences were observed between sham Reiki and actual Reiki groups, highlighting the possibility of non-specific therapeutic effects [32].

### Methodological quality and risk of bias

The methodological quality scores of the included studies ranged from 5 to 9 according to the Joanna Briggs Institute critical appraisal tools. Common methodological limitations included small sample sizes, heterogeneity in intervention protocols, limited blinding procedures, and variability in outcome measures. Additionally, one study relied primarily on aura-based biomedical measurements without conventional statistical analyses, limiting comparability across studies [19].

### The effect of Reiki practice on breast cancer patients undergoing chemotherapy

Reiki practice was associated with improvements in some symptoms in breast cancer patients undergoing chemotherapy (Table 1).

**Table 1:** Characteristics of included studies

Lead author, year, and country	QA Score	Dependent variable	Experimental Group Life Questionnaire Score Mean (SD)	Experimental Group Fatigue Score Mean (SD)	Experimental group *n*	Control Group Life Questionnaire Score Mean (SD)	Control Group Fatigue Score Mean (SD)	Control group * n*	Type of research and Reiki practice
Karaman and Tan, 2021^6^, Turkey	8	- Life Questionnaire - Fatigue	**General Well-Being Subscales** Pretest 31.90 ± 15.32 1 st measurement: 50.71 ± 16.08 2nd measurement: 62.61 ± 11.13 3rd measurement: 79.04 ± 7.92 **General Function Subscales** Pretest 44.57 ± 15.41 1 st measurement: 28.44 ± 13.83 2nd measurement: 17.33 ± 8.17 3rd measurement: 3.17 ± 3.62 **General Symptom Subscales** Pretest 48.35 ± 11.24 1 st measurement: 31.79 ± 12.86 2nd measurement: 18.38 ± 8.89 3rd measurement: 13.18 ± 1.09	1 st measurement: 5.29 ± 1.45 2nd measurement: 4.43 ± 1.25 3rd measurement: 3.42 ± 1.09	35	**General Well-Being Subscales** Pretest 37.14 ± 13.15 1 st measurement: 30.47 ± 13.54 2nd measurement: 20.00 ± 10.54 3rd measurement: 7.14 ± 7.86 **General Function Subscales** Pretest 38.81 ± 16.84 1 st measurement: 44.12 ± 13.94 2nd measurement: 55.42 ± 11.74 3rd measurement: 69.46 ± 10.04 **General Symptom Subscales** Pretest 43.29 ± 17.37 1 st measurement: 46.81 ± 16.06 2nd measurement: 47.17 ± 12.57 3rd measurement: 62.49 ± 12.89	1 st measurement: 7.03 ± 1.02 2nd measurement: 7.67 ± 0.85 3rd measurement: 8.40 ± 0.73	35	- Quasi-experimental pretest–posttest study - Reiki was administered during chemotherapy sessions and for 3 consecutive days after the first session. Six sessions were conducted in total using standard hand positions (42 min/session)
Kuril, 2020^19^, India	5	- Pain and suffering that can considerably improve the patient's quality of life - Side effects of chemotherapy on physical, mental, and emotional health	**-**	-	37	**-**	-	7	- Interventional prospective study - Prospective interventional study. Reiki was applied concomitantly with chemotherapy. Aura scans were performed before and after treatment
**Lead author, year, and country**	**QA Score**	**Dependent variable**	**Experimental Group Life Questionnaire Score ** **Mean *M* (SE)**	**Experimental Group Mood Score** ** Mean *M *(SE)**	**Experimental group ** ***n***	**Companion and control groups ** **Life Questionnaire Scores** ** Mean *M* (SE)**	**Companion and control groups ** **Mood Scores** ** Mean *M *(SE)**	**Companion and control groups ** ***n***	**Type of research and Reiki practice**
Orsak et al. 2015^17^, USA	8	-Quality of life - Mood - Symptom distress	Baseline: 103.36 (0.05) 1 st measurement: 113.36 (0.06) 2nd measurement: 113.84 (0.06) 3rd measurement: 105.53 (0.06)	Baseline: 38.28 (0.08) 1 st measurement: 24.23 (0.04) 2nd measurement: 24.48 (0.05) 3rd measurement: 29.46 (0.04)	15	**Companion:** Baseline: 110.72 (0.06) 1 st measurement: 116.53 (0.06) 2nd measurement: 120.75 (0.07) 3rd measurement: 114.48 (0.07) **Usual care:** Baseline: 99.67 (0.06) 1 st measurement: 103.54 (0.05) 2nd measurement: 101.56 (0.06) 3rd measurement: 98.79 (0.06)	**Companion:** Baseline: 47.28 (0.08) 1 st measurement: 34.38 (0.05) 2nd measurement: 18.96 (0.05) 3rd measurement: 22.26 (0.05) **Usual care:** Baseline: 52.78 (0.10) 1 st measurement: 39.20 (0.06) 2nd measurement: 35.43 (0.06) 3rd measurement: 43.84 (0.05)	**Companion**: 11 **Usual care:** 10	- Experimental study - Reiki during chemotherapy (30 min/session)
**Lead author, year, and country**	**QA Score**	**Dependent variable**	**Pairwise comparison of groups** **Comfort Scores (*p*)**	**Pairwise comparison of groups** **Well-being Scores (*p*)**	**Experimental group** *** n***	**-**	**-**	**Control group** ***n***	**Type of research and Reiki practice**
Catlin et al^32^, 2011, USA	9	- Physical well-being -Mental well-being -Physical comfort - Mental comfort	Actual Reiki versus sham Reiki placebo *p*: 0.8435 Sham Reiki placebo versus standard care *p*: 0.0027 Actual Reiki versus standard care *p*: 0.0197	Actual Reiki versus sham Reiki placebo *p*: 0.7453 Sham Reiki placebo versus standard care *p*: 0.005 Actual Reiki versus standard care *p*: 0.0051	**Actual Reiki:** 63	**-**	**-**	**Sham Reiki: 63** **Standard** **care**: 63	- Double-blind, randomized clinical controlled trial - 20-min real Reiki or sham Reiki

*QA Score*, quality appraisal score

## Discussion

This systematic review examined the effects of Reiki therapy on physical symptoms, psychological well-being, and quality of life in breast cancer patients undergoing chemotherapy. Overall, the included studies suggested that Reiki may contribute to improvements in fatigue, relaxation, comfort, mental and emotional well-being, mood, and quality of life. The findings support the growing importance of complementary therapies in symptom management. However, the findings should be interpreted cautiously because of methodological limitations, heterogeneity across studies, and the limited number of available trials.

Previous research indicates that complementary therapies play a supportive role in cancer care. It suggests that Reiki and similar energy-based therapies can have an impact on relaxation, stress reduction, emotional adjustment, and quality of life in cancer patients [11, 15, 33, 34]. These findings are consistent with the results of the studies included in this review. Karaman and Tan’s (2021) study showed that Reiki reduced psychological symptoms, particularly fatigue and general functionality sub-dimensions [6], while Orsak et al. (2015) reported significant improvements in mood [17]. Similarly, Kuril (2020) reported that Reiki practice reduced the side effects of chemotherapy on mental and emotional health, and that patients relaxed [19]. Catlin et al. (2011) reported improvements in mental comfort and mental well-being in patients with Reiki practice [32].

These findings may indicate that Reiki-based interventions contribute to relaxation and supportive care experiences during chemotherapy. An important finding of this review is that sham Reiki interventions also demonstrated improvements in comfort and well-being in one study [32]. This result suggests that factors such as therapeutic presence, human interaction, attention, relaxation response, or placebo-related effects may contribute to the benefits reported following Reiki interventions. Similar methodological challenges have been discussed in complementary therapy research, particularly for interventions involving touch, therapeutic interaction, and patient expectations [10, 34]. Therefore, it remains difficult to determine the extent to which observed effects are specifically attributable to Reiki practices versus broader supportive care mechanisms.

Within Martha E. Rogers’ Science of Unitary Human Beings framework, the findings of this review may be interpreted from a holistic nursing perspective emphasizing the dynamic interaction between individuals and their environment. Rogers describes human beings as irreducible energy fields in continuous mutual process with environmental fields, where changes in one field may influence overall well-being and pattern manifestation [22]. In the studies included in this review, Reiki interventions were associated with patient-reported improvements in relaxation, emotional comfort, mood, fatigue, and perceived well-being [6, 17, 19, 32]. These outcomes may conceptually align with Rogers’ emphasis on harmony, integrality, and patterning within the human–environment field process. In this sense, Reiki may be viewed as a supportive intervention aimed at promoting subjective experiences of balance, comfort, and well-being during chemotherapy treatment. However, Rogers’ theory was used in this review primarily as an interpretive nursing framework rather than as evidence of a specific biological or energy-based mechanism underlying Reiki effects.

Despite the limited evidence base, Reiki appeared to be a feasible and generally well-tolerated complementary intervention in the included studies. No serious adverse effects related to Reiki interventions were reported in the included studies. As part of patient-centered supportive care, Reiki may help address emotional distress and treatment-related discomfort when aligned with patient preferences and institutional policies.

## Strengths and limitations

This systematic review provides a theory-informed synthesis of current evidence regarding the effects of Reiki practice in breast cancer patients undergoing chemotherapy, integrating findings within Martha E. Rogers’ Science of Unitary Human Beings framework. The use of PRISMA 2020 guidelines, independent quality appraisal, risk-of-bias assessment, and inclusion of experimental and randomized studies strengthened the methodological rigor of the review. In addition, focusing specifically on breast cancer patients allowed a more clinically focused interpretation of outcomes within supportive oncology care.

However, several limitations should be considered. Despite the expanded search strategy and updated search period, the number of eligible studies specifically investigating Reiki interventions in breast cancer patients remained limited. The included studies had relatively small sample sizes and considerable heterogeneity in intervention protocols, session duration, practitioner experience, outcome measures, and control conditions. Some studies lacked adequate blinding procedures and relied primarily on subjective self-report outcomes, increasing the potential risk of performance and reporting bias. Furthermore, one study used aura-based biomedical assessments without conventional statistical analyses, limiting comparability with the other included studies. Variability in outcome reporting and the inconsistent presentation of effect sizes further reduced the ability to directly compare findings across studies. Additionally, the positive findings observed in sham-controlled studies raise the possibility that non-specific therapeutic factors, including attention, interaction, relaxation, and placebo-related mechanisms, may partially explain the observed benefits attributed to Reiki interventions. These limitations restrict the generalizability of the findings and prevent definitive conclusions regarding the effectiveness of Reiki in supportive cancer care.

## Implication to clinical practice and research

The findings suggest that Reiki may be considered a safe and acceptable complementary intervention to support symptom management, psychological well-being, and quality of life in breast cancer patients receiving chemotherapy. Nurses and oncology professionals may incorporate Reiki as part of holistic and patient-centered supportive care when aligned with patient preferences and institutional policies.

Future research should prioritize well-designed randomized controlled trials with larger samples, standardized intervention protocols, and blinded methodologies. Studies examining mechanisms of action, long-term outcomes, and comparative effectiveness with other supportive interventions are also needed to strengthen the evidence base for clinical integration.

## Conclusion

The findings of this systematic review suggest that Reiki may have supportive effects on symptom management, psychological well-being, comfort, and quality of life among some breast cancer patients undergoing chemotherapy. Improvements were most commonly reported in fatigue, emotional distress, stress, relaxation, and overall comfort. However, the current evidence base remains limited because of the small number of studies, modest sample sizes, heterogeneous intervention protocols, and methodological limitations across trials.

Findings from sham-controlled studies further indicate that non-specific therapeutic factors, including therapeutic attention, relaxation responses, and supportive interaction, may partly contribute to the observed outcomes. Therefore, the available evidence does not allow firm conclusions regarding the specific effectiveness or mechanisms of Reiki interventions.

Within the framework of Martha E. Rogers’ Science of Unitary Human Beings, Reiki may be conceptually interpreted as a holistic supportive practice that emphasizes harmony, comfort, and patient-centered well-being. However, this interpretation should be considered theoretical and conceptual rather than mechanistic.

Although Reiki appeared to be a feasible and generally well-tolerated complementary intervention in the included studies, stronger evidence from rigorously designed randomized controlled trials is needed before broader clinical recommendations can be made. Future studies should include larger sample sizes, standardized intervention protocols, validated outcome measures, appropriate blinding procedures, and longer follow-up periods to better clarify the potential role of Reiki in supportive oncology care.

## Data Availability

The data that support the findings of this study are available from the corresponding author upon reasonable request.
